# A prospective observational registry evaluating clinical outcomes of Radium‐223 treatment in a nonstudy population

**DOI:** 10.1002/ijc.32851

**Published:** 2020-01-21

**Authors:** Sushil K. Badrising, Rebecca D. Louhanepessy, Vincent van der Noort, Jules L.L.M. Coenen, Paul Hamberg, Aart Beeker, Nils Wagenaar, Marnix G.E.H. Lam, Filiz Celik, Olaf J.L. Loosveld, Ad Oostdijk, Hanneke Zuetenhorst, John B. Haanen, Erik Vegt, Wilbert Zwart, Andries M. Bergman, the ROTOR investigators and the Dutch Uro‐Oncology Study group (DUOS15101)

**Affiliations:** ^1^ Department of Medical Oncology Netherlands Cancer Institute Amsterdam The Netherlands; ^2^ Department of Biometrics Netherlands Cancer Institute Amsterdam The Netherlands; ^3^ Department of Medical Oncology Isala Zwolle The Netherlands; ^4^ Department of Medical Oncology Franciscus Gasthuis & Vlietland Rotterdam The Netherlands; ^5^ Department of Medical Oncology Spaarne Gasthuis Hoofddorp The Netherlands; ^6^ Department of Nuclear Medicine Ziekenhuisgroep Twente Hengelo The Netherlands; ^7^ Department of Nuclear Medicine UMC Utrecht Utrecht The Netherlands; ^8^ Department of Nuclear Medicine Deventer Hospital Deventer The Netherlands; ^9^ Department or Medical Oncology Amphia Hospital Breda The Netherlands; ^10^ Department of Nuclear Medicine Isala Zwolle The Netherlands; ^11^ Department of Nuclear Medicine Netherlands Cancer Institute Amsterdam The Netherlands; ^12^ Division of Oncogenomics Netherlands Cancer Institute Amsterdam The Netherlands

**Keywords:** prostate cancer, Radium‐223, MCRPC

## Abstract

The ALSYMPCA study established a 3.6 month Overall Survival (OS) benefit in metastatic Castration Resistant Prostate Cancer (mCRPC) patients treated with Radium‐223 dichloride (Ra‐223) over placebo. Here we report clinical outcomes of Ra‐223 treatment in a nonstudy population. In this prospective registry, patients from 20 Dutch hospitals were included prior to Ra‐223 treatment. Clinical parameters collected included previous treatments and Adverse Events. Primary outcome was 6 months Symptomatic Skeletal Event (SSE)‐free survival, while secondary outcomes included Progression‐Free Survival (PFS) and Overall Survival (OS). Of the 305 patients included, 300 were evaluable. The mean age was 73.6 years, 90% had ≥6 bone metastases and 74.1% were pretreated with Docetaxel, 19.5% with Cabazitaxel and 80.5% with Abiraterone and/or Enzalutamide. Of all patients, 96.7% were treated with Ra‐223 and received a median of 5 cycles. After a median follow‐up of 13.2 months, 6 months SSE‐free survival rate was 83%, median PFS was 5.1 months and median OS was 15.2 months. Six months SSE‐free survival rate and OS were comparable with those reported in ALSYMPCA. “Previous Cabazitaxel treatment” and “bone‐only metastases” were independent predictors of a shorter and longer PFS, respectively, while above‐median LDH and “bone‐only metastases” were independent predictors of shorter and longer OS, respectively. Toxicity was similar as reported in the ALSYMPCA trial. These results suggest that in a nonstudy population, Ra‐223 treatment is well‐tolerated, equally effective as in the ALSYMPCA population and that patients not previously treated with Cabazitaxel benefit most from Ra‐223.

## Abstract 2

**What's new?**

The ALSYMCA phase III trial reported a survival benefit from Radium‐223 treatment in patients with metastatic castration‐resistant prostate cancer (mCRPC). These patients, however, were not treated previously with newer life‐prolonging drugs, raising questions about whether the ALSYMCA population reflects current populations of mCRPC patients. In this prospective study, men with mCRPC who were pretreated with newer generation drugs prior to receiving Radium‐223 experienced improvements in disease‐free and overall survival that were comparable to those reported in the ALSYMPCA study. Prior treatment with cabazitaxel independently predicted shorter progression‐free survival. The findings suggest that, overall, Radium‐233 is effective in men with mCRPC.

**Abbreviations**
95% CI: 95% Confidence Interval
Abi: abiraterone
AE: adverse event
ALP: alkaline phosphatase
ALSYMPCA: alpharadin in symptomatic prostate cancer
BPI: brief pain inventor
Cab: cabazitaxel
Doc: docetaxel
EBRT: external‐beam radiation therapy
ECOG: Eastern Cooperative Oncology Group
EMA: European Medicines Agency
Enz: enzalutamide
HR: hazard ratio
IQR: Interquartile range
KM: Kaplan–Meier
LDH: lactate dehydrogenase
mCRPC: metastatic castration‐resistant prostate cancer
OS: overall survival
PCWG3: Prostate Cancer Workgroup 3
PFS: progression‐free survival
PRAC: Pharmacovigilance Risk Assessment Committee
PSA: prostate‐specific antigen
Ra‐223: radium 223
RECIST: response evaluation criteria in solid tumors
SAS: statistical analysis system
SSE: symptomatic skeletal event

## Introduction

Prostate cancer is the second most common cancer in men worldwide, with over 1 million newly diagnosed cases each year.^1^ At presentation, approximately 11% of patients have bone metastases,^2^ while approximately 70% of metastatic prostate cancer patients develop bone metastases during the course of their disease.^3^ Bone metastases have a detrimental impact on quality of life.^4^ These metastases are the most common cause of cancer‐related pain, pathological fractures, compression of the spinal cord, vertebral instability and hypercalcemia.^5^

Until 2013, bone directed treatment of symptomatic metastases was limited to beta‐emitting radionuclides, external beam‐radiation therapy (EBRT), bisphosphonates, denosumab and surgery.^6^ Although these therapies are effective for pain palliation and prevention, no Overall Survival (OS) benefit was established.^7^, ^8^ This changed with the introduction of Radium‐223 dichloride (*Xofigo®*; Ra‐223), a targeted alpha therapy that selectively binds to areas of increased bone turnover. In 2013, the ALSYMPCA phase III trial reported a survival benefit of 3.6 months in metastatic Castration‐Resistant Prostate Cancer (mCRPC) patients treated with Ra‐223 compared to placebo, rendering Ra‐223 the only radionuclide treatment with a survival benefit.^6^

Over recent years, the treatment options for mCRPC patients have expanded.^9^ Although, patients previously treated with Docetaxel (Doc) as well as patients not treated with Doc were included in ALSYMPCA, none of these patients had been treated with the newer life‐prolonging agents Abiraterone (Abi), Enzalutamide (Enz) and Cabazitaxel (Cab). These newer generation drugs became available after accrual of the ALSYMPCA trial was completed.^10^, ^11^, ^12^, ^13^ This raises the question whether the results of ALSYMPCA are representative for present patients treated with Ra‐223. Therefore, we conducted a prospective registry of Ra‐223 treated mCRPC patients in the Netherlands. The primary goal of this registry was to assess the 6 months symptomatic skeletal event (SSE)‐free survival rate in a nonstudy population, while OS, progression‐free survival (PFS) and safety were secondary outcomes.

## Study Design and Patients

In this noninterventional, multicenter, prospective, observational registry, patients aged 18 years or older with progressive mCRPC and scheduled for Ra‐223 treatment were included in 20 hospitals in the Netherlands. This registry was approved by local medical ethics committees. Obtaining signed Informed Consent was not required, but patients had to provide oral consent and written approval for the documentation and use of their identifiers. Patients received Ra‐223 at the treating physician's discretion. There were no other inclusion and exclusion criteria or stopping rules. During Ra‐223 treatment, patients were evaluated at the outpatient clinic every 4 weeks during treatment, where ECOG performance, adverse events (AE) and clinical lab assessments were documented. Radiological evaluation during and after Ra‐223 treatment and frequency of follow‐up visits was at the physician's discretion. All patients scheduled to be treated with Ra‐223 were included in our analysis. Clinical data were collected from the medical records after completion of Ra‐223 treatment.

### Procedures and data

Using an electronic case‐report form, we recorded multiple baseline characteristics, efficacy assessments, AE (graded according to Common Terminology Criteria for Adverse Events v. 4.0^14^) and SSE during Ra‐223 treatment (defined as the time from inclusion to first need for EBRT to relieve skeletal symptoms, new pathological fractures, spinal cord compression, or tumor‐related orthopedic surgical intervention). Patients were considered symptomatic when they used analgesics regularly or were treated with EBRT for cancer‐related bone pain in the previous 12 weeks, which is the same definition as used in ALSYMPCA.^15^

Progression‐free survival (PFS) was calculated from the date of first Ra‐223 treatment to the date of confirmed progression. Patients were considered progressive in case of clinical progression (defined as clinical signs of progression), radiological progression (according to RECIST v. 1.1),^16^ started with subsequent treatment or death, all in line with PCWG3 recommendations.^17^ OS was calculated from the date of the first Ra‐223 cycle to the date of death or censored at last follow‐up. PSA and Alkaline Phosphatase (ALP) declines from baseline during Ra‐223 treatment of ≥30, ≥50 and ≥90% were evaluated as best response. Time to ALP progression was defined as an increase of ≥25% from baseline at ≥12 weeks, in patients with no decrease from baseline, or as an increase of ≥25% above the nadir in patients with an initial decrease from baseline. Time to subsequent treatment was defined as time from last cycle of Ra‐223 until the start of any systemic life‐prolonging antiprostate cancer treatment. The treating physician provided reasons for discontinuation of Ra‐223.

### Sample size and statistical analysis

The dual endpoints of the study are 6 months SSE‐free survival and reduction of pain as measured by the Brief Pain Inventory (BPI). ALSYMPCA reported an SSE‐free survival rate at 6 months of 78%. We calculated that if the SSE‐free 6 months survival rate in our population is similar to that in the ALSYMPCA population, with 300 patients, we can estimate it with a 95% confidence interval of 5% points above and below. In particular, we expect to have over 95% power to show that the 6 months SSE‐free survival rate on Ra‐223 lies statistically significantly above 70%. In parallel to these considerations, we looked at the power to detect a decrease in pain score when comparing patient‐reported outcomes (PRO) while on radium treatment with baseline. Of each of the 300 patients, we expected an average of 4.5 measurements, bringing the expected number of measurements to 1,350. However, for the sake of the power calculation, we restricted ourselves to a very basic comparison of only two measurements per patients: pain after 6 months and pain at baseline. For this comparison, simulations show that under a wide range of assumptions concerning the initial distribution of pain scores over the patients, with 300 patients, we have more than 95% power to detect (in a paired t‐test) an average decrease in pain as small as one point on BPI pain scale. The pain response on Ra‐223 therapy will be presented in a separate publication, along with other PROs.

In line with PCWG3 recommendations,^17^ survival and progression were evaluated using Kaplan–Meier (KM) estimates. The log‐rank test was used in univariate survival analysis to identify variables that could predict OS and PFS. Factors with *p* values ≤0.10 were included in a multivariate model for survival rate by Cox proportional‐hazard analysis. Statistical analyses were conducted using Statistical Analysis System (SAS) statistical software (SAS Institute Inc., Chicago, IL) and R (R Foundation for Statistical Computing, Vienna, Austria).^18^

### Data availability

The data that support the findings of our study are available from the corresponding author upon reasonable request.

## Results

### Patient characteristics

**Figure 1:**
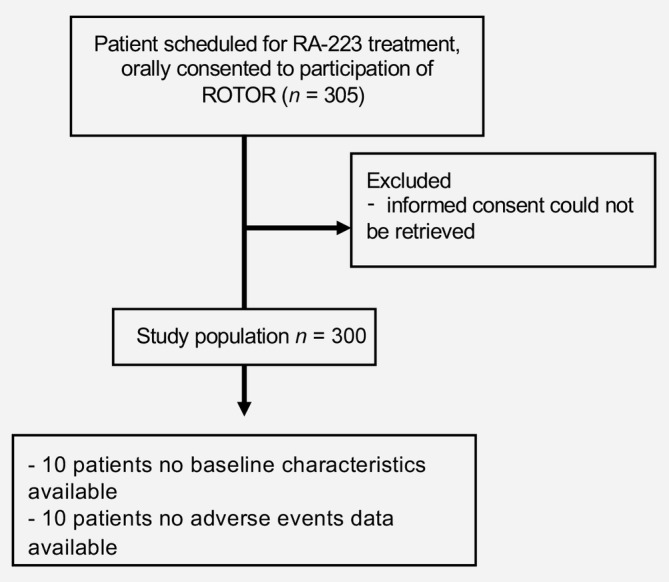
Consort diagram

Between February 2015 and March 2018, 305 patients from 20 Dutch hospitals were enrolled in the ROTOR. The median follow‐up was 13.2 months (95% confidence interval [CI] 12.1–14.4 months). Five patients were excluded because written approval to use identifiers was not obtained or not properly stored according to guidelines. Therefore, 300 patients were evaluable (Fig. 1). Out of these 300 patients, 10 had no baseline data available and from 10 no AE data were collected. Baseline patient characteristics are summarized in Table 1. Practically all patients had an ECOG performance score of 0–1, all patients had two or more bone metastases and 19.6% of the patients were asymptomatic prior to RA‐223 treatment.

**Table 1:** Patient and treatment characteristics

Patient demographics	Median [IQR], number of patients (%) or value (*n* = patients evaluable)
Age, years	73.6 [46.3–91.5]
ECOG performance status	*n* = 279
0–1	264 (94.6)
2	15 (5.3)
≥3	0
Symptomatic patients	131(80.4)
Asymptomatic patients	32 (19.6)
Gleason	*n* = 251
≤7	87 (34.9)
8	67 (26.9)
≥9	95 (38.2)
Metastatic sites	*n* = 290
Bone	287 (99.0)
Lymph nodes	84 (29.0)
Visceral organs	0 (0)
No. of bone metastases	*n* = 272
0–1	0 (0)
2–6	21 (7.7)
>6	246 (90.4)
Super scan	5 (1.8)
Laboratory values	
PSA, μg/l	72.3 [25.0–175.0]
Hemoglobin, g/dl	12.6 [11.3–13.4]
ALP, U/L	138 [85–248]
ALP ≥220 U/l	81 (27.9)
LDH, U/L	225.0 [192–296]
Albumin, g/L	42 [38–44]
Calcium, mmol/ml	2.35 [2.26–2.43]
Testosterone, nmol/l	0.5 [0.45–0.50]
Previous lines of systemic life‐prolonging treatments	*n* = 300
0	34 (11.3)
1	104 (34.7)
2	96 (32.0)
3	50 (16.7)
4	13 (4.3)
5	3 (1.0)
Specific previous treatments	*n* = 266
Abiraterone and or Enzalutamide	214 (80.5)
Docetaxel	197 (74.1)
Cabazitaxel	52 (19,5)
Radiotherapy 12 weeks prior to treatment	26 (8.7)
Concomitant medication	*n* = 294
Bisphosphonates	49 (16.7)
Denosumab	63 (24.4)

### Previous, concomitant and RA‐223 treatment

For 11.3% of the patients, Ra‐223 was the first treatment line, 34.7% received 1 line of systemic treatment prior to Ra‐223, while 54% of the patients received 2 or more lines prior to Ra‐223 (Table 1). Of the patients treated with life‐prolonging therapy prior to Ra‐223, 80.5% were treated with Abi and/or Enz, 74.1% were treated with Doc and 19.5% were treated with Cab. All patients treated with Cab had previously been treated with Doc. EBRT within 12 weeks prior to Ra‐223 was received by 8.7% of patients. Forty‐one percent of the patients were treated with bisphosphates or denosumab. Of the 300 evaluable patients, 290 received at least one cycle of Ra‐223, while the median number of cycles was 5 and 54.7% of patients received at least 5 Ra‐223 cycles (Table 2). Reported reasons for Ra‐223 treatment discontinuation included, six cycles completed (46.3%), symptomatic progression (35%), no PSA response (20.7%) and radiological progression (16%; Table 2).

**Table 2:** Outcomes of radium‐223 treatment

Outcome variables	Median [IQR], No. of patients (%) or 95% CI
No. of radium‐223 cycles	
Median number of cycles	5.0 [3–6]
0	10 (3.3)
1–2	40 (13.3)
3–4	86 (28.7)
5–6	161 (53.7)
>6	3 (1.0)
ALP decline	*n* = 255
≥30%	122 (47.8)
≥50%	56 (22.0)
≥90%	1 (0.4)
Time to ALP progression, Months	6.3 (6.0–6.6)
PSA decline	*n* = 256
≥30%	16 (6.3)
≥50%	11 (4.3)
≥90%	3 (1.2)
Reason for Radium‐223 discontinuation	
Six cycles completed	139 (46.3)
Symptomatic progression	105 (35.0)
No PSA response	62 (20.7)
Radiological progression	48 (16.0)
Intolerance	44 (14.7)
Death	8 (2.7)
Other/Reason unknown	9 (3.0)
Symptomatic skeletal event during Radium‐223 treatment	
Total SSE	58 (19.3)
Pathological fractures	7 (2.3)
Radiotherapy	33 (11.0)
Spinal cord compression	17 (5.7)
Bone surgery	1 (0.3)
Time to first SSE, months	*Median not reached*
Progression‐free survival (months)	
Whole population	5.1 (4.5–5.8)
Patients >30% PSA decline	10.4 (6.6–14.2)
Patients >30% ALP decline	6.2 (5.1–7.3)
Patients with bone‐only metastases	5.5 (4.9–6.0)
Symptomatic patients	4.3 (3.3–5.3)
Asymptomatic patients	5.9 (4.9–6.9)
Patients not treated with cabazitaxel	5.2 (4.5–5.9)
Patients treated with cabazitaxel	4.2 (3.5–4.8)
Overall survival (months)	
Whole population	15.2 (12.8–17.6)
Patients >30% PSA decline	21.0 [14.7–27.2)
Patients >30% ALP decline	19.1 (13.5–24.6)
Symptomatic patients	13.4 (9.5–17.3)
Asymptomatic patients	*Median not reached*
Time to subsequent treatment, months	5.9 (4.1–7.7)
Hospital admission during Radium‐223 treatment	82 (28.1)

Abbreviations: ALP, serum alkaline phosphatase; PSA, serum prostate‐specific antigen; SSE, symptomatic skeletal event.

### Clinical outcomes

**Figure 2:**
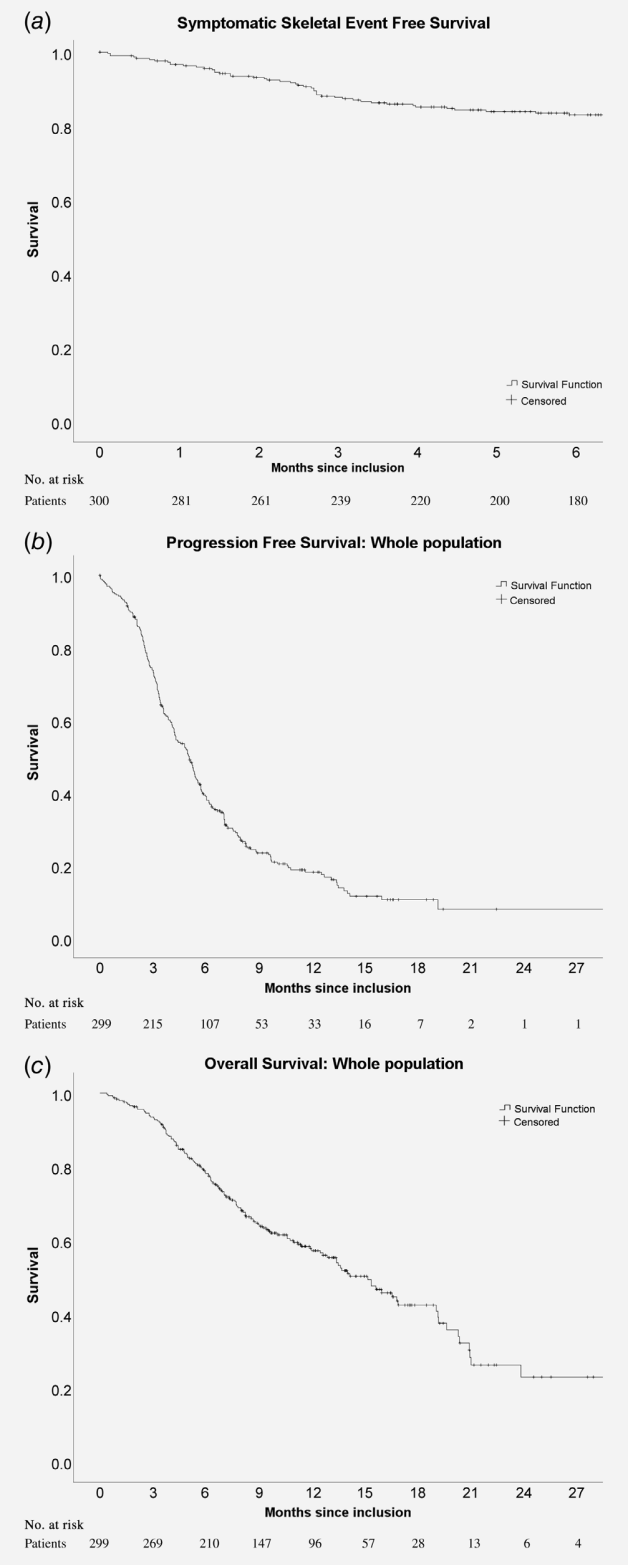
Kaplan–Meier curves of (*a*) symptomatic skeletal event‐free survival, (*b*) progression‐free survival of the whole population and (*c*) overall survival of the whole population

The primary outcome of our study, 6 months SSE‐free survival was 83%, which is 5% higher than the 78% reported in ALSYMPCA (Fig. 2 *a*). During Ra‐223 treatment, 58 patients (19.3%) experienced an SSE (Table 2); 2.3% of these SSE were pathological fractures, 5.7% spinal cord compressions, 11% EBRTs and 0.3% tumor‐related orthopedic surgical intervention. After a median follow‐up of 13.2 months (95% CI 12.1–14.4), PFS was 5.1 months (Fig. 2 *b*) and OS was 15.2 months (Fig. 2 *c*; Table 2).

PSA and ALP declines of ≥50% were observed in 4.3 and 22.0% of patients, respectively (Table 2). Patients with a PSA decline >30% had median PFS and OS of 10.4 and 21.0 months, respectively. Patients with ALP declines >30% had median PFS and OS of 6.2 and 19.1 months, respectively (Table 2). Both PSA and ALP responses were related with longer than median PFS and OS, while ALP responses were more frequent. Time to ALP progression was 6.3 months (95% CI 6.0–6.6; Table 2). Although asymptomatic patients were infrequent, these patients had better PFS compared to symptomatic patients, 5.9 and 4.3 months, respectively (Table 2). Symptomatic patients had an OS of 13, 4 months, while there were not enough events to calculate OS in asymptomatic patients. (Supporting Information Fig. S1*a*; Table 2). PFS in patients previously treated with Cab was 4.2 month, while Cab naïve patients had a PFS of 5.2 months (Table 2; Supporting Information Fig. S2*d*). During Ra‐223 treatment 28.1% of the patients were hospitalized and median time from first Ra‐223 treatment until the start of subsequent treatment was 5.9 months (95% CI 4.1–7.7; Table 2).

### Univariate and multivariate analysis affecting PFS and OS

Univariate and multivariate analysis of factors associated with PFS and OS are summarized in Table 3. According to univariate analysis, previous Doc, previous Cab, elevated ALP, elevated LDH and higher Gleason score, were associated with shorter PFS, while higher serum calcium levels, higher hemoglobin, bone‐only metastases, >30% ALP decline and >30% PSA decline and number of Ra‐223 cycles were associated with longer PFS. However, the multivariate analysis only confirmed previous use of Cab and bone‐only metastases as independent predictors of a shorter and longer PFS, respectively.

**Table 3:** Univariate and multivariate analysis of factors affecting progression‐free survival (PFS) and overall survival (OS)

Variable	PFS						OS					
	Univariate			Multivariate			Univariate			Multivariate		
	HR	95% CI	*p*	HR	95%	*p*	HR	95% CI	*p*	HR	95%	*p*
Age + 1 years	0.98	0.97–1.0	0.010	1.01	0.98–1.04	0.628	1.00	0.98–1.02	0.750			
ECOG + 1	1.10	0.84–1.42	0.47				1.31	0.94–1.85	0.114			
Calcium	0.41	0.14–1.18	0.098	1.50	0.25–9.14	0.658	0.36	0.14–0.89	0.027	0.61	0.06–6.51	0.680
Log ALP	1.29	1.15–1.46	<0.001	1.22	0.96–1.55	0.106	1.51	1.31–1.73	<0.001	1.25	0.94–1.65	0.124
>30% ALP decline	0.58	0.45–0.75	<0.001				0.68	0.48–0.97	0.028			
Log PSA	1.05	0.99–1.12	0.081	0.98	0.89–1.08	0.663	1.19	1.09–1.29	<0.001	1.07	0.94–1.22	0.301
>30% PSA decline	0.36	0.21–0.61	<0.001				0.39	0.18–0.83	0.005			
Log LDH	1.84	1.50–2.26	<0.001	1.28	0.82–1.00	0.284	3.09	2.43–3.93	<0.001	2.99	1.85–4.84	<0.001
Hb + 1	0.89	0.82–0.96	0.004	0.98	0.84–1.14	0.779	0.76	0.69–0.85	<0.001	0.94	0.77–1.15	0.534
Gleason + 1	1.27	1.12–1.44	<0.001	1.05	0.87–1.26	0.62	1.16	0.99–1.36	0.071	0.92	0.75–1.14	0.451
Number of metastases	1.01	1.00–1.01	0.03	1.02	1.01–1.03	0.003	1.18	0.91–1.51	0.211			
Only bone metastases	0.57	0.43–0.75	<0.001	0.31	0.19–0.49	<0.001	0.49	0.35–0.69	<0.001	0.21	0.13–0.46	<0.001
Line nr	1.04	0.91–1.19	0.59				1.22	1.03–1.45	0.023	1.38	0.89–2.13	0.148
Abi and/or Enz	0.78	0.56–1.08	0.128				1.26	0.80–1.98	0.314			
Docetaxel	1.41	1.03–1.94	0.031	1.33	0.81–2.22	0.248	1.40	0.92–2.13	0.113			
Cabazitaxel	1.44	1.04–1.99	0.026	1.89	1.01–3.51	0.046	2.31	1.58–3.38	<0.001	1.46	0.64–3.34	0.371
Denosumab	0.99	0.67–1.27	0.605				0.69	0.44–1.08	0.105			
Bisphosphonates	0.95	0.68–1.34	0.767				1.15	0.76–1.76	0.505			
Symptomatic	1.29	0.72–2.34	0.394				1.15	0.74–1.80	0.535			
Number of Ra223 cycles	0.63	0.58–0.69	<0.001				0.60	0.56–0.64	<0.001			

All values are at baseline. Age + 1 Y, HR per year age difference; ECOG + 1, HR per every ECOG‐point increase; Line nr, HR per every additional treatment‐line. Calcium: HR per 1.0 mmol/l increase of calcium; Log ALP: HR per every doubling of Alkaline phosphatase; >30% ALP decline: >30% ALP decline during treatment from baseline; Log PSA: HR per every doubling of PSA; >30% PSA decline: >30% PSA decline during treatment from baseline; Log LDH: HR per every doubling of Lactate dehydrogenase; Hb + 1: HR per every point (g/dL) of hemoglobin increase; Gleason+1: HR per every point of Gleason score increase; No of metastases: HR per increase of number of metastases ranging from 0 to 1. 2–6. >6 or superscan. Symptomatic: Patients were considered symptomatic when they used analgesics regularly or were treated with EBRT for cancer‐related bone pain in the previous 12 weeks; No of Ra223 cycles: every additional cycle after the first Ra‐223 cycle.

Univariate analysis suggested an association between shorter OS and line of treatment, previous Cab, elevated ALP and elevated LDH, while higher serum calcium, bone‐only metastases, >30% ALP decline, number of Ra‐223 cycles and >30% PSA decline were associated with a longer OS. Only elevated LDH and bone‐only metastases were independent predictors in a multivariate Cox model of shorter and longer OS, respectively.

### Tolerability

Adverse event (AE) was collected from 290 patients (Supporting Information Table S1). Grade 3 anemia was observed in 18.6% of the patients, but no Grade 4. Grade 3 thrombocytopenia was observed in 3.1% of the patients and Grade 4 in 1%. Grade 3 neutropenia was observed in 2.4% and Grade 4 in 0.3%. The most common reported nonhematologic AE was fatigue (61.4%). The majority of these patients had Grade 1–2 fatigue (55.5%). Other common nonhematologic AEs (all grades) were nausea (31%) and diarrhea (28.6%).

## Discussion

When ALSYMPCA was conducted, no life‐prolonging treatments apart from Doc were available. Patients not fit for, or refusing chemotherapy had no other treatment options than participation in the ALSYMPCA trial, while nowadays these patients are treated with less toxic second‐generation androgen receptor‐antagonists.^19^, ^20^ As a result, only 11% of patients received Ra‐223 as a first‐line treatment in the present cohort, while 80.5% of patients were treated with Abi or Enz prior to Ra‐223. Moreover, 54% of patients received two or more lines of treatment prior to Ra‐223 treatment. This suggests that with the introduction of new life‐prolonging treatment options, Ra‐223 is now used in more pretreated patients than in ALSYMPCA.

Although patients in the registry were predominantly treated with Ra‐223 in second and later lines, and patients in the ALYMPCA population were treated in first or second line, OS in the registry is comparable to OS in the treatment arm of ALSYMPCA (15.2 months and 14.9, respectively).^6^ The comparable OS might be attributed to strict patient selection for Ra‐223 treatment in real‐life, but also to effective subsequent treatments. Better patient selection is reflected by a lower frequency of ECOG ≥2 scores (5 and 13%, respectively), lower baseline PSA levels (PSA 72.3 and 146 μg/l, respectively) and lower baseline ALP levels (ALP 128 and 211 U/l, respectively) in the nonstudy cohort when compared to the ALSYMPCA population. Univariate regression analysis in our cohort suggests that higher PSA and higher ALP are associated with shorter OS, confirming reports from previous studies.^21^, ^22^

The rate of ≥30% ALP declines in our cohort was similar to what was reported in ALSYMPCA (47.8 and 46.8%, respectively). In the present cohort, a >30% decrease of ALP from baseline was associated with a longer median PFS and OS when compared to the entire cohort. This favorable outcome was also reflected in univariate analysis of PFS and OS. This is in agreement with the results of a post hoc analysis of ALSYMPCA, where a significant decline in risk of death in patients with an ALP decline after 12 weeks was reported.^6^, ^23^ A >30% PSA decline from baseline was associated with an even more favorable PFS and OS in univariate analysis when compared to the entire cohort. In contrast to our findings, a post hoc analysis of ALSYMPCA reported no correlation between PSA response and OS.^23^

Compared to ALSYMPCA, patients in the present cohort had more Grade 3 anemia (18 and 11%, respectively). Other hematological AEs were similarly frequent as in ALSYMPCA. The most common nonhematological AEs in the present cohort and in ALSYMPCA were nausea (27 and 36%, respectively), diarrhea (27.7 and 25%, respectively) and fatigue (61.4 and 26%, respectively). The cause of the significant difference in fatigue between the studies is unclear, since there are no major differences between baseline ECOG‐performance score and baseline hemoglobin between our cohort and ALSYMPCA. The more advanced and pretreated stage of nonstudy mCRPC patients treated with Ra‐223 might account for the differences found. However, the differences are mainly in the occurrence of Grade 0–2 fatigue (55 and 22%, respectively), while Grade 3 (5.9 and 4%, respectively) and Grade 4 fatigue (0 and 1%, respectively) are comparable.

An update of safety in ALSYMPCA, 3 years after first injection revealed no new long‐term complications or safety concerns.^6^, ^24^ Also, the present cohort raises no new short‐term safety concerns, apart from those already reported in ALSYMPCA. However, the Pharmacovigilance Risk Assessment Committee (PRAC) from the European Medicines Agency (EMA) recently advised restricting the use of Ra‐223 to third‐line treatment or to patients with no other treatment options.^25^, ^26^ This recommendation was based on higher mortality and fracture rates in patients treated with Abi and Ra‐223 in the ERA‐223 trial.^27^ It was concluded that the mortality was not the result of the interaction between Ra‐223 and Abi, but due to Ra‐223 treatment alone. In our real‐life population, the majority of patients were treated with Ra‐223 in second or third line, while the rate of SSE was comparable to ALSYMPCA, where patients were treated in first or second line. This could not be attributed to differences in the use of denosumab or bisphosphonates, which was approximately 40% in both studies. Therefore, our study does not support the advice to treat patients in later line with Ra‐223 for safety reasons.

In univariate analysis of our cohort, more systemic anticancer treatment prior to Ra‐223 did not affect PFS. However, line of Ra‐223 treatment was associated with OS, which is obviously the consequence of more advanced disease in later lines of treatment. There was no association between prior Abi or Enz treatment and PFS or OS, but both in univariate and multivariate cox‐regression analysis, previous Cabazitaxel treatment was associated with a less favorable PFS and OS. The association between prior chemotherapy and shorter survival has been reported in retrospective studies, while Alva *et al*. reported that prior treatment with Abi or Enz had no negative effect on OS, which agrees with our findings.^28^, ^29^ Bone‐only metastases was associated with favorable PFS and OS in both univariate and multivariate cox‐regression analysis. However, in ALSYMPCA, patients with a single lymphadenopathy of <3 cm in the short‐axis diameter were included.^6^ In the current study, no data was collected on the extension of extraosseous metastases. Consequently, no new cutoff for the maximum number of lymph nodes involved, in order to benefit from Ra‐223 can be suggested.

The main limitation of our study was its nonrandomized nature. Moreover, dates of radiological assessments prior, during and after Ra‐223 treatment were not prescribed but at the discretion of the treating physician. Another limitation of our study was that there are likely different criteria between the participating centers to select patients for Ra‐223 treatment. This might be the result of the absence of reliable data to base selection on. However, these differences in patient selection reflect the nonstudy nature of the population.

In conclusion, this prospective registry of Ra‐223 treatment in a nonstudy mCRPC population, suggests that Ra‐223 is safe and effective. Moreover, efficacy in the nonstudy population seems comparable with the less treated ALSYMPCA population. Moreover, the data of this nonstudy cohort suggests that patients not previously treated with Cab, have a favorable outcome. These findings need confirmation.

## Conflict of interest

Bergman participated in Advisory Boards of Janssen Pharma, Bayer, Sanofi and Astellas, received speaking fees from Astellas, Bayer, Jansen Pharma and Astellas and received a research grants from Sanofi and Astellas, not related to our study. Zwart participated in Advisory Boards of Astellas and received research grants of Astellas and AstraZeneca. Haanen has provided consultation, attended advisory boards and/or provided lectures for AIMM, Amgen, AZ/Medimmune, Bayer, BMS, GSK, Ipsen, Merck Serono, MSD, Novartis, Pfizer, Roche/Genentech, Sanofi, Seattle Genetics for which NKI received honoraria. He also received grant support from Bayer, BMS, MSD, Novartis, Neon Therapeutics and Pfizer. All remaining authors have declared no conflicts of interest.

## Supporting information


**Data S1**: Supporting Information
