# Supplementary material for: A prospective observational registry evaluating clinical outcomes of Radium‐223 treatment in a nonstudy population
Source: Int J Cancer. 2020 Jan 21;147(4):1143–51. doi: 10.1002/ijc.32851 (PMC7383569; doi:10.1002/ijc.32851)
Supplement: Supplementary file 1 — Data S1: Supporting Information [file IJC-147-1143-s001.pdf]

Supplementary Material

A Prospective Observational Registry Evaluating Clinical Outcomes of Radium-223 Treatment in a Non-study Population

Authors:

Sushil K Badrising[1*], Rebecca D Louhanepessy[1*], Vincent vd Noort[2], Jules LLM Coenen[3], Paul Hamberg[4], Aart Beeker[5], Nils Wagenaar[6], Marnix Lam[7], Filiz Celik[8], O.J.L Loosveld[9], Ad Oostdijk[10], Hanneke Zuetenhorst[5], John B Haanen[1], Erik Vegt[11], Wilbert Zwart[12#], Andries M Bergman[1,12#] on behalf of the ROTOR investigators and the Dutch Uro-Oncology Study group (DUOS15101).

Table of contents

1. Supplementary Table
2. Supplementary Figure

Supplementary table 1 Adverse events

**Most common hematologic**	**Present at baseline No. of Patients (%) (*n*=290)**	**During treatment No. of Patients (%)**
Anemia		
Grade 3	51 (17.6)	54 (18.6)
Grade 4	0 (0)	0 (0)
Thrombocytopenia		
Grade 3	3 (1.0)	9 (3.1)
Grade 4	1 (0.3)	3 (1.0)
Neutropenia		
Grade 3	2 (0.3)	7 (2.4)
Grade 4	0 (0)	1 (0.3)
Most common non-hematologic		
Nausea (all grades)	7 (2.4)	90 (31.0)
Diarrhea (all grades)	1 (0.3)	83 (28.6)
Fatigue (all grades)	47 (16.2)	178 (61.4)
Grade 1-2	40 (13.8)	161 (55.5)
Grade 3	7 (2.4)	17 (5.9)
Grade 4	0 (0)	0 (0)

Supplementary Figure 1

A **Overall Survival: symptomatic patients**

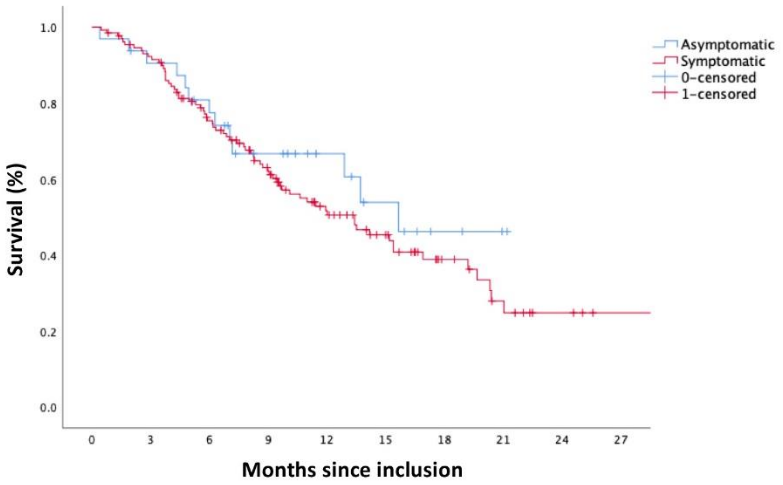

No. at Risk

Not Sympt.	32	27	22	15	10	6	2	0	0	0
Sympt.	131	117	88	65	43	30	15	8	3	0

B **Progression Free survival: previous cabazitaxel treatment**

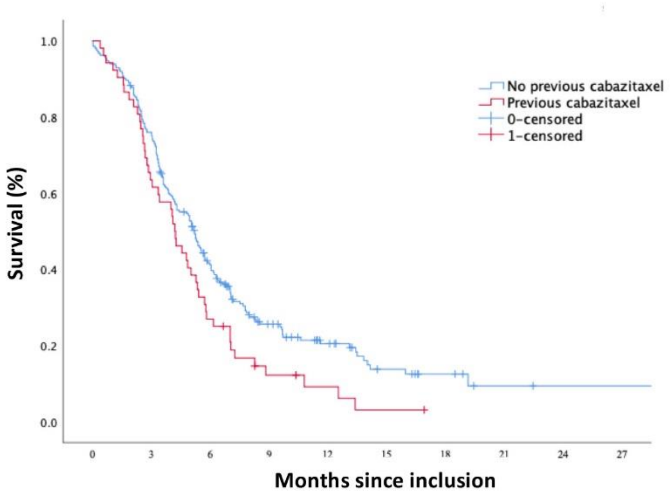

No. at Risk

No CAB	213	160	79	38	22	10	5	1	1	1
CAB	52	32	13	4	2	0	0	0	0	0

Legend

Figure 1a: Kaplan-Meier curves of Overall survival in symptomatic and asymptomatic patients.

Figure 1b: Kaplan-Meier curves of progression Free survival in patients previously treated with Cabazitaxel
